# Acrylic-assisted immobilization of Cu_2_O–Ag domains on a 3D loofah monolith for adsorption-assisted visible-light photodecolorization

**DOI:** 10.1039/d6ra07776e

**Published:** 2026-10-22

**Authors:** Xuan Thang Dam, Thi Thu Giang Pham, Thuy Van Ngo, Trong Tuan Nguyen, Truong Xuan Vuong

**Affiliations:** a Faculty of Chemical Technology, Hanoi University of Industry Vietnam thangdx@haui.edu.vn; b Faculty of Garment Technology and Fashion Design, Hanoi University of Industry Vietnam; c Faculty of Natural Sciences and Technology, TNU-University of Science Tan Thinh Ward Thai Nguyen City 24000 Vietnam

## Abstract

Immobilizing visible-light-responsive photocatalysts within recoverable porous architectures requires a balance among active-phase retention, interfacial accessibility, and photon exposure. Here, Cu_2_O–Ag-containing domains were immobilized on a three-dimensional loofah scaffold through an acrylic-assisted strategy to construct a recoverable Cu_2_O–Ag@ACR/LF-3D monolith integrating a lignocellulosic sorptive framework, a polymeric immobilization interface, and visible-light-responsive domains. FTIR, XRD, SEM-EDX mapping, and UV-vis diffuse reflectance analyses confirmed incorporation of the acrylic phase, retention of crystalline Cu_2_O, spatial distribution of Cu- and Ag-containing domains, and an apparent optical band gap of 1.99 eV. Control experiments showed that the pronounced dye removal was associated predominantly with the intact monolith rather than adsorption, direct photolysis, or leached species alone. Response surface methodology identified optimum conditions at pH 5.94, an initial dye concentration of 68.07 mg L^−1^, and a monolith dosage of 0.71 g, yielding a predicted decolorization efficiency of 98.14% and an experimentally validated value of 96.43%. TOC decreased by approximately 62% after 180 min, indicating substantial but incomplete mineralization, while reactive-species trapping supported the participation of ˙O_2_^−^, h^+^, and ˙OH, with stronger contributions from ˙O_2_^−^ and h^+^-associated pathways. The monolith remained readily recoverable, although efficiency decreased to 63.62% after five reuse cycles. Post-cycle SEM-EDX and AAS revealed surface evolution and measurable Cu release, identifying active-phase retention, particularly Cu retention, as a principal materials-stability limitation.

## Abstract 2

A recoverable Cu_2_O–Ag@ACR/LF-3D monolith integrates adsorption and visible-light photocatalysis, achieving 96.43% dye decolorization and ∼62% TOC reduction under optimized conditions. Generated with Google Gemini.

## Graphical abstract

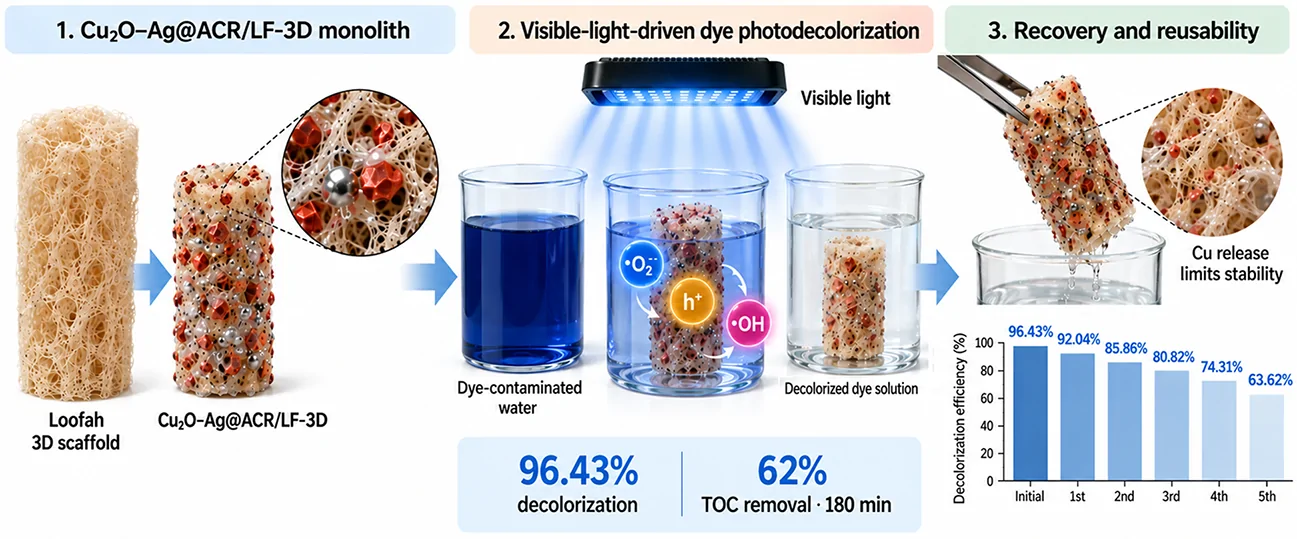

## 1. Introduction

Textile dyeing and finishing processes generate persistent colorants that are often poorly biodegradable and difficult to remove by conventional biological treatment.^1–3^ In light-driven treatment systems, performance depends not only on semiconductor activity but also on dye-surface affinity, photon penetration, accessibility of photoactive domains, and interfacial mass transfer.^4^ These coupled factors have motivated the development of structured visible-light-responsive materials that combine photochemical functionality with sorptive accessibility, structural integrity, and straightforward recovery.

Lignocellulosic biomass is an attractive platform for such structured materials because of its hierarchical porosity, surface oxygen functionalities, and suitability for monolithic shaping.^5,6^ Biomass-derived architectures can simultaneously provide sorptive interfaces and supports for immobilized photoactive phases, thereby increasing dye availability near accessible reactive domains.^7–9^ Compared with slurry photocatalysts, these structured configurations simplify solid–liquid separation and repeated handling. Their performance, however, can be limited by restricted light penetration, transport resistance, non-uniform active-phase deposition, and structural or surface changes during repeated aqueous operation.^10–12^

Loofah sponge (*Luffa cylindrica*) is particularly suitable as a three-dimensional scaffold because its interconnected fibrous channels facilitate liquid permeation while providing a readily recoverable monolithic framework.^13–16^ Its lignocellulosic surface contains hydroxyl and other oxygen-containing functionalities that can contribute to dye association through hydrogen bonding, electrostatic interactions, and related interfacial forces.^17–22^ Loofah-based materials have therefore been investigated as adsorbents, biocarriers, and structured supports for water-treatment applications.^23–27^ Nevertheless, pristine loofah provides limited control over the distribution and retention of deposited functional phases, and surface modification, catalytic loading, carbonization, and composite strategies have been explored to improve active-phase anchoring and repeated-use performance.^28–30^

Cuprous oxide (Cu_2_O) is a visible-light-responsive semiconductor with a narrow band gap of approximately 2.0–2.2 eV, but rapid charge-carrier recombination and the instability of near-surface Cu(i) species can restrict its performance during prolonged aqueous irradiation.^8,9^ Coupling Cu_2_O with Ag has been widely investigated to modify its optical and interfacial charge-transfer behavior.^31–35^ Although plasmonic enhancement and interfacial carrier transfer are frequently invoked for Ag-containing semiconductor systems, these effects depend strongly on particle size, spatial contact, composition, morphology, and irradiation conditions and therefore cannot be inferred from decolorization data alone.^36^ Most Cu_2_O–Ag photocatalysts have nevertheless been studied as dispersed particles, nanoscale suspensions, or conventional coated substrates, whereas their incorporation into recoverable porous biomass-polymer monoliths remains comparatively less explored.^37,38^

A key materials challenge is therefore to immobilize Cu_2_O–Ag-containing domains without excessively compromising pore accessibility or photon exposure. Structured supports can facilitate catalyst recovery and limit particle loss, but excessive or non-uniform coatings may mask active sites, attenuate incident light, and increase transport resistance.^39,40^ Acrylic-based matrices offer a practical route for consolidating lignocellulosic scaffolds and promoting adhesion between organic supports and deposited functional phases.^41–44^ Polymer-assisted coating strategies can also improve mechanical integrity and processability, while polar groups at the polymer–water interface may contribute to local dye association.^45–50^ What remains unclear is whether Cu_2_O–Ag-containing domains can be immobilized within a biomass-polymer monolith while preserving porous accessibility and visible-light response, and whether such an architecture can retain sufficient active-phase stability during repeated aqueous operation. The unresolved issue is therefore not simply photocatalytic activity, but the balance among active-phase immobilization, interfacial dye accessibility, photon exposure, and metal retention within a recoverable structured material.

In this work, a recoverable Cu_2_O–Ag@ACR/LF-3D monolith was constructed through sequential *in situ* formation of Cu_2_O–Ag-containing domains followed by acrylic-assisted immobilization on a three-dimensional loofah scaffold. FTIR, XRD, SEM-EDX mapping, and UV-vis diffuse reflectance spectroscopy were used to examine the structural, morphological, elemental, and optical characteristics of the material. Dark-adsorption and photolysis controls were employed to distinguish sorptive uptake from irradiation-stage photodecolorization, while apparent pseudo-first-order rate constants were used only as comparative kinetic descriptors. Response surface methodology was applied to define the operating region, with ANN used as a complementary nonlinear assessment rather than as an independent validation tool. Reusability, post-cycle surface evolution, elemental distribution, and dissolved Cu/Ag release were further evaluated. The study therefore focuses on the structure–function–stability relationship of a recoverable Cu_2_O–Ag/biomass-polymer monolith, rather than on decolorization efficiency alone.

## 2. Materials and methods

### 2.1. Materials

Luffa cylindrica was collected in Phu Tho Province, Vietnam. Silver nitrate (AgNO_3_, ≥99.8%), copper(ii) sulfate pentahydrate (CuSO_4_·5H_2_O, ≥99%), and ascorbic acid (≥99%) were purchased from Sigma-Aldrich, Germany. Calcium hydroxide (Ca(OH)_2_) was obtained from a local supplier in Vietnam. Sodium hydroxide (NaOH) and hydrogen peroxide (H_2_O_2_) were of analytical grade and used as received. A waterborne acrylic polyol resin supplied by Yu-Ta Resin Chemical Co., Ltd, Taiwan, with a solids content of 70 ± 2% and a hydroxyl value of 70–75 mg per KOH per g, was used as the immobilization/binding phase. Synozol Navy Blue K-GF (*λ*_max_ = 610 nm) was used as the model reactive dye. Deionized water was used throughout the experiments.

### 2.2. Sequential *in situ* formation and acrylic-assisted immobilization of the Cu_2_O–Ag@ACR/LF-3D monolith

The collected loofah was air-dried, manually cleaned by removing the seeds and outer rind, and cut into discs 15–20 mm in diameter and 5–8 mm in thickness. The discs were treated for 60 min with 0.17% (w/v) lime water containing 4% (v/v) H_2_O_2_ at a liquid-to-loofah ratio of 15 : 1. After treatment, the discs were thoroughly rinsed with deionized water until the wash water reached pH 6.5–7.0 and then air-dried. The resulting pretreated three-dimensional loofah scaffold was denoted LF-3D.

LF-3D discs (approximately 0.50 g) were immersed in 100 mL of CuSO_4_ solution (0.0075 mol L^−1^) and stirred at 25 ± 2 °C. Subsequently, 100 mL of NaOH solution (0.015 mol L^−1^) was added dropwise to induce *in situ* precipitation of Cu(OH)_2_ on the scaffold. A 10 mL portion of ascorbic acid solution (0.05 mol L^−1^) was then added dropwise as the reducing agent, and stirring was continued for 20–30 min. During this stage, the deposited phase gradually changed from deep blue to yellow–orange or brick red, consistent with the formation of Cu_2_O.

Without isolating the Cu_2_O/LF-3D intermediate, 100 mL of AgNO_3_ solution (0.0075 mol L^−1^) was introduced under light-shielded conditions, corresponding to an overall Cu : Ag molar feed ratio of 1 : 1. During Ag^+^ addition, the remaining 10 mL of ascorbic acid solution (0.05 mol L^−1^) was added intermittently to promote reduction and deposition of Ag-containing domains. Subsequently, 15 mL of the waterborne acrylic dispersion was introduced as an immobilization/binding phase to assist retention of the Cu_2_O–Ag-containing domains on the LF-3D scaffold. The mixture was stirred for an additional 10–20 min.

The resulting grayish-brown monolith was removed from the reaction medium, thoroughly washed with deionized water until the wash water reached pH 6.5–7.0, and dried at 60 °C for 24 h. The final material was denoted Cu_2_O–Ag@ACR/LF-3D. The sequential fabrication route is illustrated in Fig. 1.

**Fig. 1:**
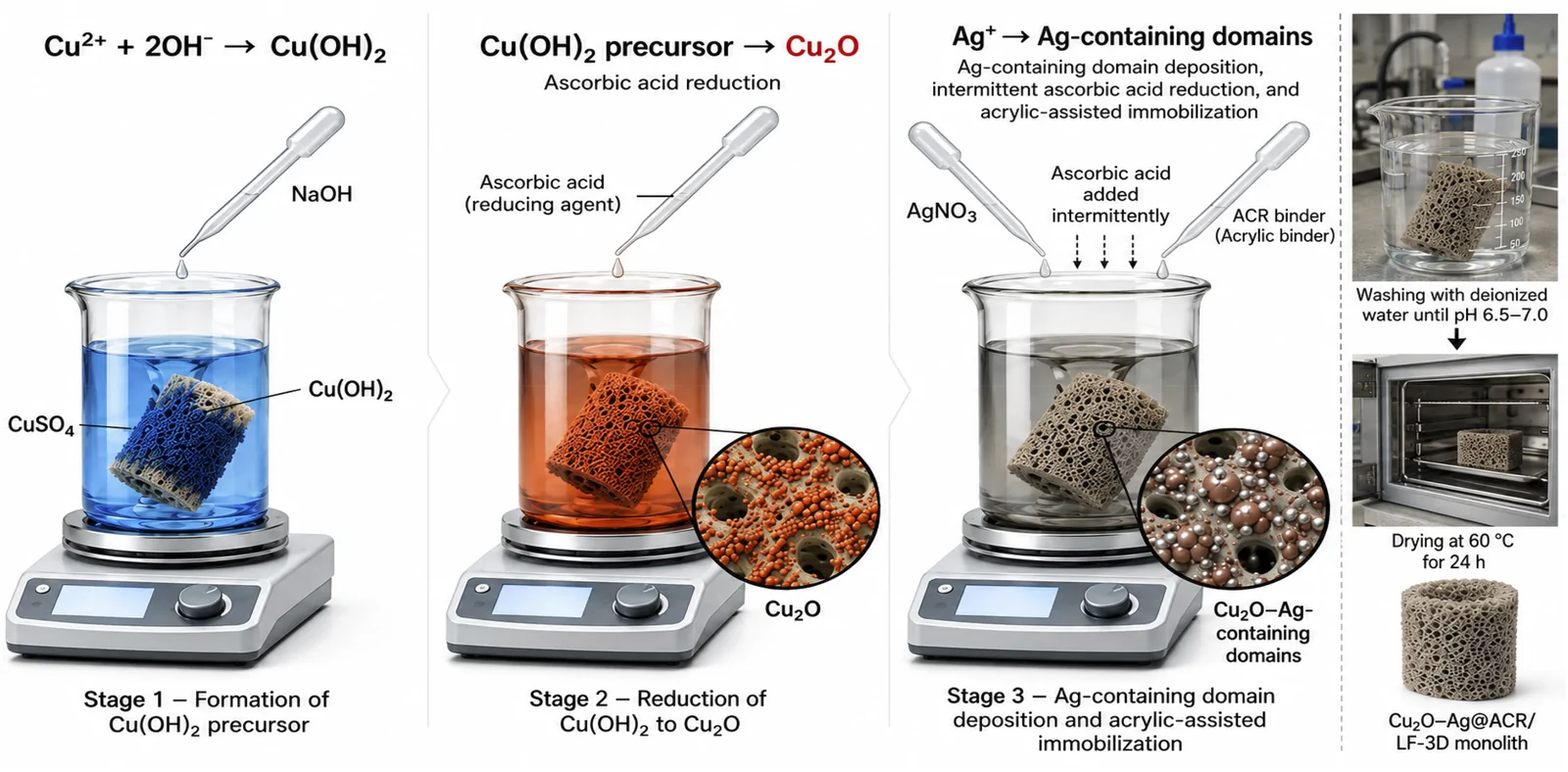
Sequential *in situ* formation of Cu_2_O–Ag-containing domains and their acrylic-assisted immobilization on the three-dimensional loofah scaffold to obtain the Cu_2_O–Ag@ACR/LF-3D monolith.

### 2.3. Characterization

Fourier-transform infrared (FTIR) spectra were recorded in attenuated total reflectance mode using a Nicolet iS10 spectrometer (Thermo Scientific, USA) over the wavenumber range of 4000–500 cm^−1^ at a resolution of 4 cm^−1^ with 32 scans. X-ray diffraction (XRD) patterns were collected using a D8 Advance diffractometer (Bruker AXS, Karlsruhe, Germany) with Cu Kα radiation (*λ* = 0.154 nm) over a 2*θ* range of 10–80°. Surface morphology, local elemental composition, and elemental distribution were examined by scanning electron microscopy coupled with energy-dispersive X-ray spectroscopy (SEM-EDX) and elemental mapping using a JSM-6510LV instrument (JEOL, Japan) operated at accelerating voltages of 5–15 kV. SEM-EDX analyses were performed on selected regions of the fresh and used monoliths to evaluate morphological evolution and the persistence and spatial distribution of Cu- and Ag-containing domains. Because EDX provides local, semi-quantitative surface information, the measured elemental fractions were interpreted only as representative of the analyzed regions and not as bulk metal contents. N_2_ adsorption–desorption measurements were performed for LF-3D, ACR/LF-3D, and Cu_2_O–Ag@ACR/LF-3D at 77.35 K using a TriStar II Plus analyzer. Prior to analysis, the samples were degassed at 100 °C overnight. The Brunauer–Emmett–Teller (BET) method was used to estimate the N_2_-accessible specific surface area, while the total pore volume was obtained from the adsorption data at high relative pressure.

Diffuse-reflectance UV-vis spectra were recorded using a UV-2450 spectrophotometer (Shimadzu, Kyoto, Japan) equipped with an integrating sphere, with BaSO_4_ used as the reference, over the wavelength range of 200–800 nm. The reflectance data were transformed using the Kubelka–Munk function, *F*(*R*_∞_), and the apparent optical band gap, *E*_g_, was estimated from Tauc plots of [*F*(*R*_∞_)*hν*]^2^*versus hν*, assuming a direct allowed transition.

### 2.4. Batch adsorption tests

Batch adsorption experiments were conducted to assess the affinity of the Cu_2_O–Ag@ACR/LF-3D monolith toward Synozol Navy Blue K-GF (*λ*_max_ = 610 nm). Dye solutions were prepared in deionized water, and the initial pH was adjusted using 0.1 mol L^−1^ HCl or NaOH. A known mass of the monolith was contacted with a fixed volume of dye solution in capped Erlenmeyer flasks at 25 ± 2 °C and 150 rpm. At predetermined time intervals, aliquots were withdrawn, filtered through 0.22 µm membranes, and analyzed by UV-vis spectrophotometry at 610 nm. Dye concentrations were determined from external calibration curves with *R*^2^ = 0.999. All experiments were performed in triplicate.

The adsorption capacities at time *t*, *q*_*t*_, and at equilibrium, *q*_e_, were calculated as:^19^1
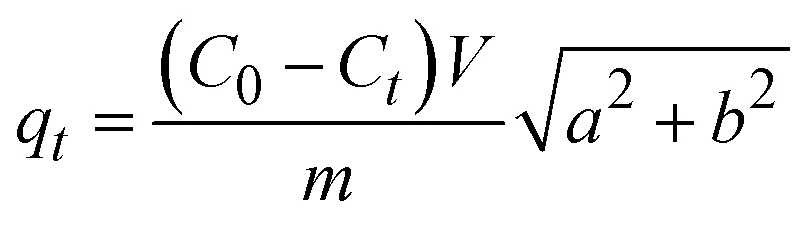
2
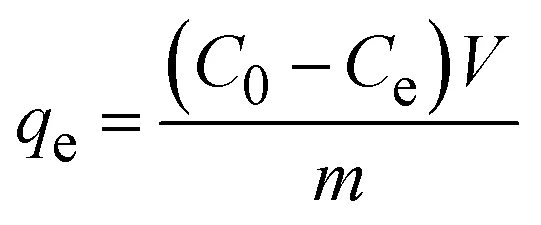
where *C*_0_, *C*_*t*_, and *C*_e_ are the initial, time-dependent, and equilibrium dye concentrations (mg L^−1^), respectively; *V* is the solution volume (*L*); and *m* is the monolith mass (*g*). Detailed adsorption kinetic and isotherm analyses are provided in the SI.

### 2.5. Visible-light photocatalysis

#### 2.5.1. Photoreaction system and experimental setup

Visible-light irradiation (*λ* ≥ 420 nm) was provided by a vertically mounted 300 W short-arc xenon lamp (TOPTION, China) equipped with a visible-light cutoff filter. The distance between the lamp outlet and the liquid surface was maintained at 4–5 cm. The lamp position, power setting, reactor placement, and irradiation geometry were kept constant throughout all experiments to maintain consistent irradiation conditions.

Batch photodecolorization experiments were conducted in 250 mL conical flasks containing 200 mL of dye solution. The Cu_2_O–Ag@ACR/LF-3D monolith was fully immersed in the solution and positioned centrally beneath the incident light. Unless otherwise stated, experiments were performed without mechanical agitation at 25 ± 2 °C. Aliquots were withdrawn from a fixed position in the bulk solution to minimize sampling-related variability.

#### 2.5.2. Photodecolorization procedure and sampling

Synozol Navy Blue K-GF solutions were prepared at the required initial concentration, *C*_0_, and adjusted to the selected pH. A Cu_2_O–Ag@ACR/LF-3D monolith of known mass was immersed in 200 mL of dye solution and maintained in the dark for 30 min before irradiation. The dye concentration measured after this dark-contact period was denoted 
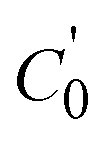
 and used as the effective initial concentration for the irradiation stage.^10,51^

Visible-light irradiation was then initiated, and aliquots were withdrawn at predetermined time intervals and filtered through 0.22 µm membranes. When necessary, aliquots were appropriately diluted with deionized water prior to UV-vis analysis at 610 nm to ensure that the measured absorbance remained within the validated linear calibration range. Dye concentrations were determined from the external calibration curve (*R*^2^ ≥ 0.999), and the original concentrations were back-calculated using the corresponding dilution factors. All photodecolorization experiments were performed in triplicate, and the results are reported as mean ± standard deviation (*n* = 3).

To assess organic-carbon removal beyond chromophore attenuation, total organic carbon (TOC) was determined for the Cu_2_O–Ag@ACR/LF-3D system. Samples were collected immediately after the 30 min dark-contact period, before irradiation (TOC_0_), and after 180 min of visible-light irradiation (TOC_180_). TOC reduction was calculated as 
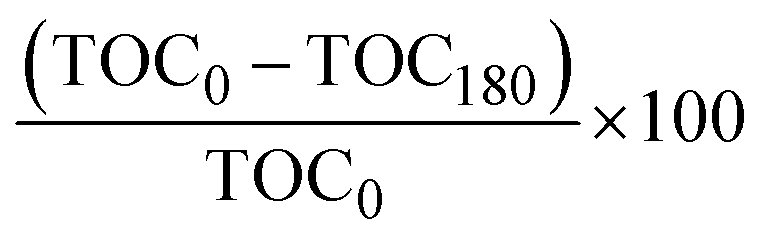
.

#### 2.5.3. Dark adsorption, controls, and kinetic analysis

Dark-adsorption and photolysis controls were conducted under otherwise identical experimental conditions using, respectively, dye solution containing the Cu_2_O–Ag@ACR/LF-3D monolith without irradiation and dye solution without the monolith under visible-light irradiation.^2,3^ Additional visible-light controls included LF-3D, ACR/LF-3D, and a leachate-only system. For the leachate-only control, the Cu_2_O–Ag@ACR/LF-3D monolith was removed from the corresponding dye-treatment system before visible-light irradiation, and the resulting solution was irradiated without the monolith under otherwise identical conditions. This control was used to assess whether species released from the monolith could account for a substantial fraction of the observed decolorization.

The irradiation-stage decolorization kinetics were described using an apparent pseudo-first-order model:^9^3
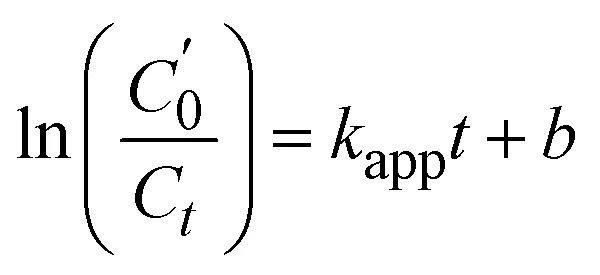
where *C*_*t*_ is the dye concentration at irradiation time *t*, 
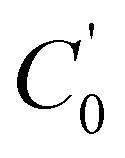
 is the dye concentration measured after the dark-contact period, *k*_app_ is the apparent rate constant (min^−1^), and *b* is the fitted intercept. Model adequacy was evaluated using *R*^2^ and residual analysis. The fitted *k*_app_ values were interpreted only as comparative kinetic descriptors under the specified irradiation conditions and not as intrinsic photocatalytic rate constants.

The overall removal efficiency, *H*_total_, and the irradiation-stage decolorization efficiency, *H*_photo_, were calculated as:4
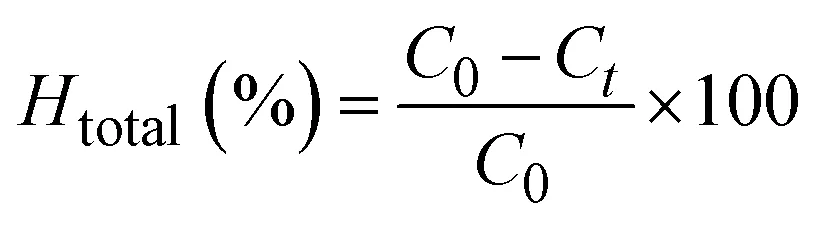
5
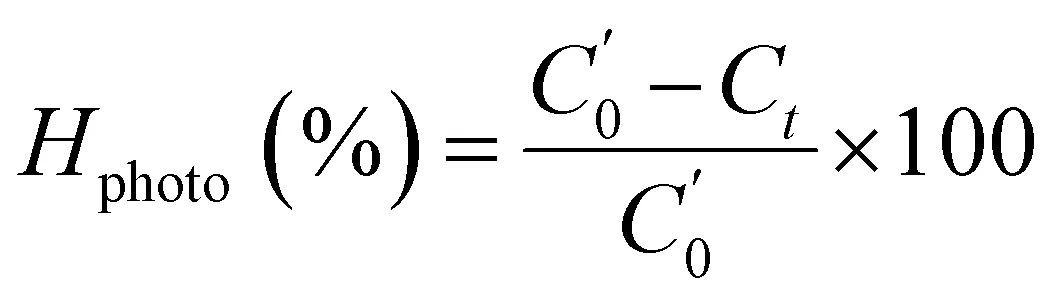
where *C*_0_ is the initial dye concentration before dark contact. *H*_total_ represents the combined contribution of pre-irradiation adsorption and subsequent visible-light treatment, whereas *H*_photo_ represents the irradiation-stage decolorization referenced to 
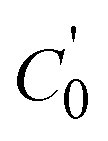
.

Reactive-species trapping experiments were conducted using Cu_2_O–Ag@ACR/LF-3D to assess the involvement of photogenerated reactive species. Isopropanol (IPA, 1 mM), *p*-benzoquinone (BQ, 1 mM), and triethanolamine (TEOA, 1 mM) were independently used as scavengers for hydroxyl radicals (˙OH), superoxide radicals (˙O_2_^−^), and photogenerated holes (h^+^), respectively. Each trapping system was maintained in the dark for 30 min before visible-light irradiation. The dye concentration measured after the dark-contact period was taken as the corresponding irradiation-stage initial concentration 
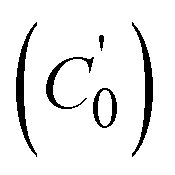
, and the decolorization efficiency after 180 min was calculated relative to the individual 
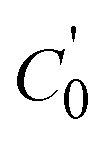
 of each trapping system.

### 2.6. Reusability and stability

The reusability of the Cu_2_O–Ag@ACR/LF-3D monolith was evaluated through an initial treatment run followed by five consecutive reuse cycles under fixed operating conditions. In each run, the same monolith specimen was contacted with the dye solution in the dark for 30 min and subsequently irradiated with visible light (*λ* ≥ 420 nm) for a fixed irradiation time, t*. The cycling experiments were performed in triplicate, and the results are reported as mean ± standard deviation (*n* = 3).

After each run, the monolith was removed, rinsed with deionized water followed by an ethanol/water mixture (1 : 1, v/v), dried at 50 °C for 2 h, and reused in the subsequent cycle. Activity retention was calculated as:6
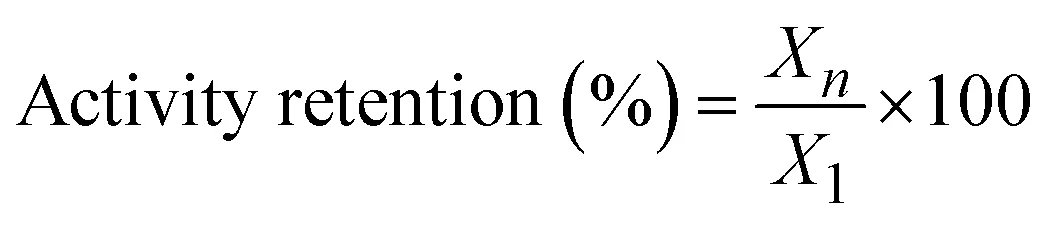
where *X*_*n*_ and *X*_1_ are the decolorization efficiencies measured at t* for reuse cycle *n* and the initial treatment run, respectively.^9,32,33^

To assess active-phase stability during repeated operation, the corresponding treated solutions were filtered through 0.22 µm membranes and analyzed for dissolved Cu and Ag by atomic absorption spectroscopy (AAS) using an iCE 3500 instrument (Thermo Scientific, Germany).

### 2.7. RSM optimization and complementary ANN assessment

Visible-light decolorization efficiency, *H*_total_, was optimized using a three-factor, three-level Box–Behnken design (BBD) within the response surface methodology framework.^52–54^ The investigated factors were solution pH (*A*, 5.5–6.5), initial dye concentration (*B*, *C*_0_, 65–75 mg L^−1^), and monolith dosage (*C*, 0.65–0.75 g). Seventeen experimental runs, including five center points, were fitted to a second-order polynomial model:7
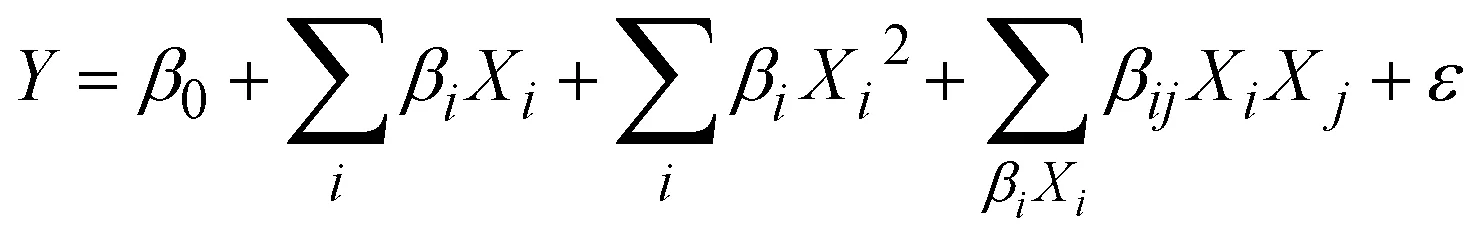
where *Y* is *H*_total_; *β*_0_, *β*_*i*_, *β*_*ii*_, and *β*_*ij*_ are the intercept, linear, quadratic, and interaction coefficients, respectively; and *ε* is the residual error. Model adequacy was evaluated by analysis of variance (ANOVA), lack-of-fit testing, *R*^2^, adjusted *R*^2^, predicted *R*^2^, and residual diagnostics. Numerical optimization was used to identify the operating conditions that maximized *H*_total_, followed by experimental confirmation.

A feed-forward multilayer perceptron artificial neural network (ANN) was used as a complementary nonlinear assessment,^55^ with the three BBD factors as input variables and *H*_total_ as the output. The standardized network comprised two hidden layers containing 16 and 8 neurons, respectively, and was trained using the ReLU activation function and Adam optimizer. Model-data agreement was evaluated using *R*^2^, root mean square error (RMSE), mean absolute error (MAE), and maximum absolute error. Because the ANN was developed using the same 17-run BBD dataset, it was used only as a complementary consistency assessment and not as an independent predictive validation model.

## 3. Results and discussion

### 3.1. Structural, morphological, and optical characteristics of Cu_2_O–Ag@ACR/LF-3D

#### 3.1.1. Structural and optical characterization

The structural and optical characteristics of LF-3D and the Cu_2_O–Ag-containing materials were examined by FTIR, XRD, UV-vis diffuse reflectance spectroscopy, and Tauc analysis, as summarized in Fig. 2.

**Fig. 2:**
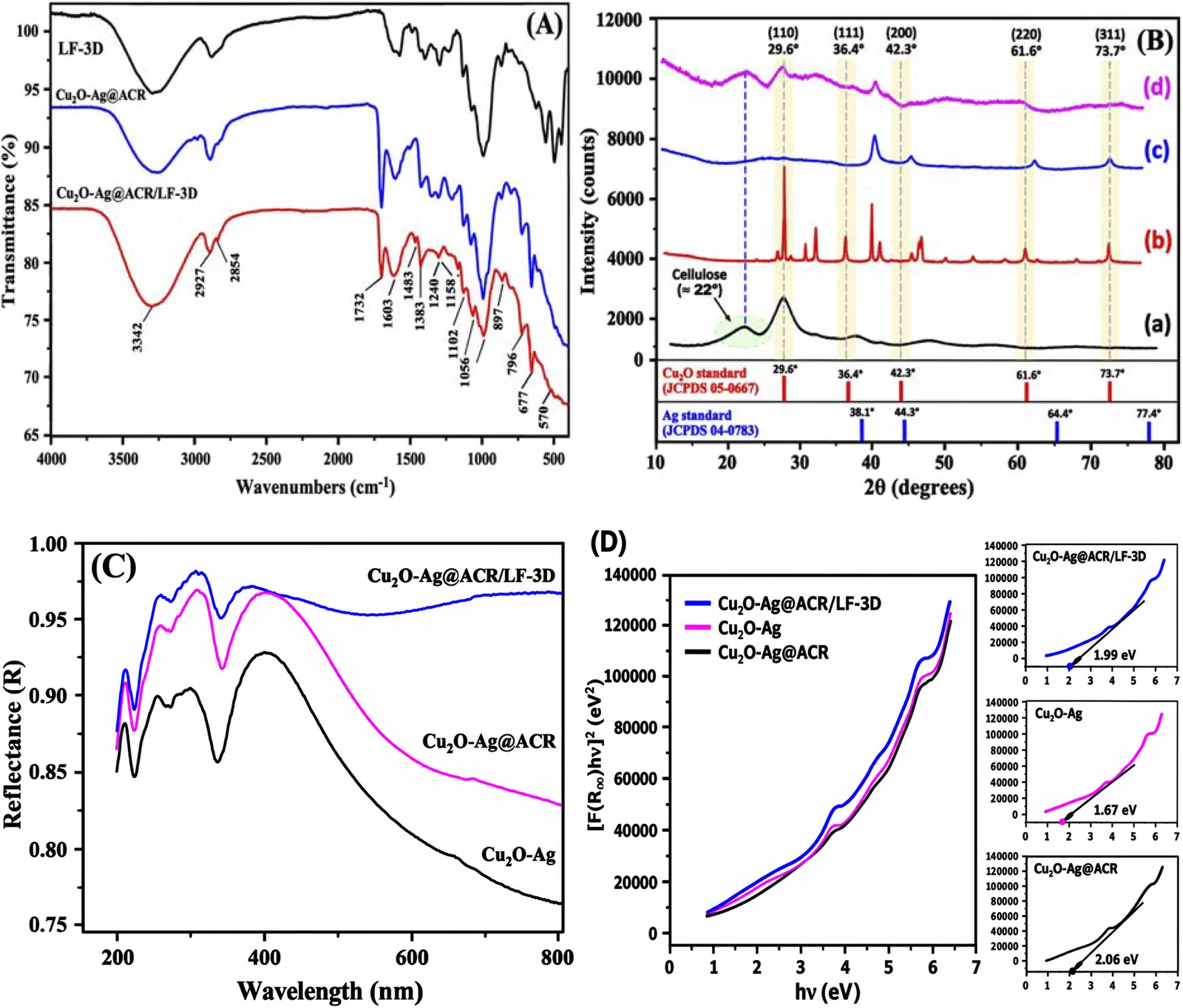
Structural and optical characterization of Cu_2_O–Ag-based materials: (A) FTIR spectra; (B) XRD patterns of (a) LF-3D, (b) Cu_2_O–Ag, (c) Cu_2_O–Ag@ACR, and (d) Cu_2_O–Ag@ACR/LF-3D. Reference peak positions for Cu_2_O (JCPDS No. 05-0667) and Ag (JCPDS No. 04-0783) are shown beneath the experimental diffractograms for comparison; (C) UV-vis diffuse reflectance spectra; and (D) Tauc plots with linear extrapolations.

The FTIR spectrum of LF-3D shows the characteristic features of a lignocellulosic scaffold, including a broad O–H stretching band at approximately 3342 cm^−1^, C–H stretching in the 2872–3027 cm^−1^ region, and an intense C–O-related band at 1026–1102 cm^−1^.^17,18,21^ After incorporation of the acrylic phase, Cu_2_O–Ag@ACR and Cu_2_O–Ag@ACR/LF-3D exhibit a more pronounced carbonyl absorption at approximately 1726 cm^−1^ together with enhanced C–O/C–O–C features in the 1200–1310 cm^−1^ region. These spectral changes support incorporation of the acrylic component within the immobilized composite. Features retained in the low-wavenumber region are consistent with Cu–O-containing domains and support preservation of the Cu_2_O component after immobilization.^9,32,33^

The XRD pattern of LF-3D displays the broad diffraction feature characteristic of semicrystalline cellulose near 2*θ* ≈ 22°. The Cu_2_O–Ag-containing sample exhibits distinct reflections at approximately 29.6°, 36.4°, 42.3°, 61.6°, and 73.7°, assigned to the (110), (111), (200), (220), and (311) planes, respectively, of cubic cuprite Cu_2_O.^8,31,32^ These reflections remain detectable after acrylic-assisted immobilization and integration with LF-3D, although their relative intensities decrease because of the contribution of the predominantly amorphous organic matrix. No clearly resolved reflections attributable to CuO are observed within the detection limits of the present measurements, supporting retention of the cuprite phase during composite fabrication. Metallic Ag reflections are expected near approximately 38.1° and 44.3° in related Cu_2_O–Ag systems;^8,31,32^ however, these regions are affected by neighboring Cu_2_O reflections and background contributions. Therefore, metallic Ag cannot be unambiguously identified from the present XRD data alone. SEM-EDX and elemental mapping confirm the presence and spatial distribution of Ag as an element, but do not determine its oxidation state or crystalline phase.

The diffuse reflectance spectra show distinct optical responses following acrylic-assisted immobilization and integration with the LF-3D scaffold (Fig. 2C). These differences may reflect contributions from the Cu_2_O–Ag-containing domains, acrylic phase, lignocellulosic matrix, and wavelength-dependent light scattering. The reflectance data were therefore transformed using the Kubelka–Munk function to estimate apparent optical band gaps.

Assuming a direct allowed transition, linear extrapolation of the corresponding Tauc plots yielded apparent optical band gaps of 1.67, 2.06, and 1.99 eV for Cu_2_O–Ag, Cu_2_O–Ag@ACR, and Cu_2_O–Ag@ACR/LF-3D, respectively (Fig. 2D). The apparent band gap increased following acrylic incorporation and decreased slightly after immobilization on the LF-3D scaffold. These values are consistent with visible-light absorption and are comparable to those reported for Cu_2_O-containing heterostructures.^56,57^ For comparison, a Cu_2_O/Ag-coated biochar composite was reported to exhibit an optical band gap of approximately 2.05 eV, compared with approximately 2.19 eV for the corresponding Ag-free Cu_2_O-containing material.^10^ However, the observed differences should not be interpreted as direct evidence of changes in the intrinsic electronic structure, because wavelength-dependent scattering from the acrylic and porous lignocellulosic matrices may affect the Kubelka–Munk transformation and Tauc extrapolation. The reported values are therefore regarded as apparent optical band gaps.

Overall, FTIR supports incorporation of the acrylic phase, while XRD confirms retention of crystalline Cu_2_O after immobilization. Together with the apparent band-gap estimates from diffuse reflectance analysis, these results demonstrate that the Cu_2_O–Ag@ACR/LF-3D architecture preserves the cuprite phase while maintaining a visible-light-responsive optical profile.

#### 3.1.2. Morphology and elemental distribution

The surface morphology and elemental distribution of the Cu_2_O–Ag@ACR/LF-3D monolith were examined by SEM, EDX, and elemental mapping (Fig. 3).

**Fig. 3:**
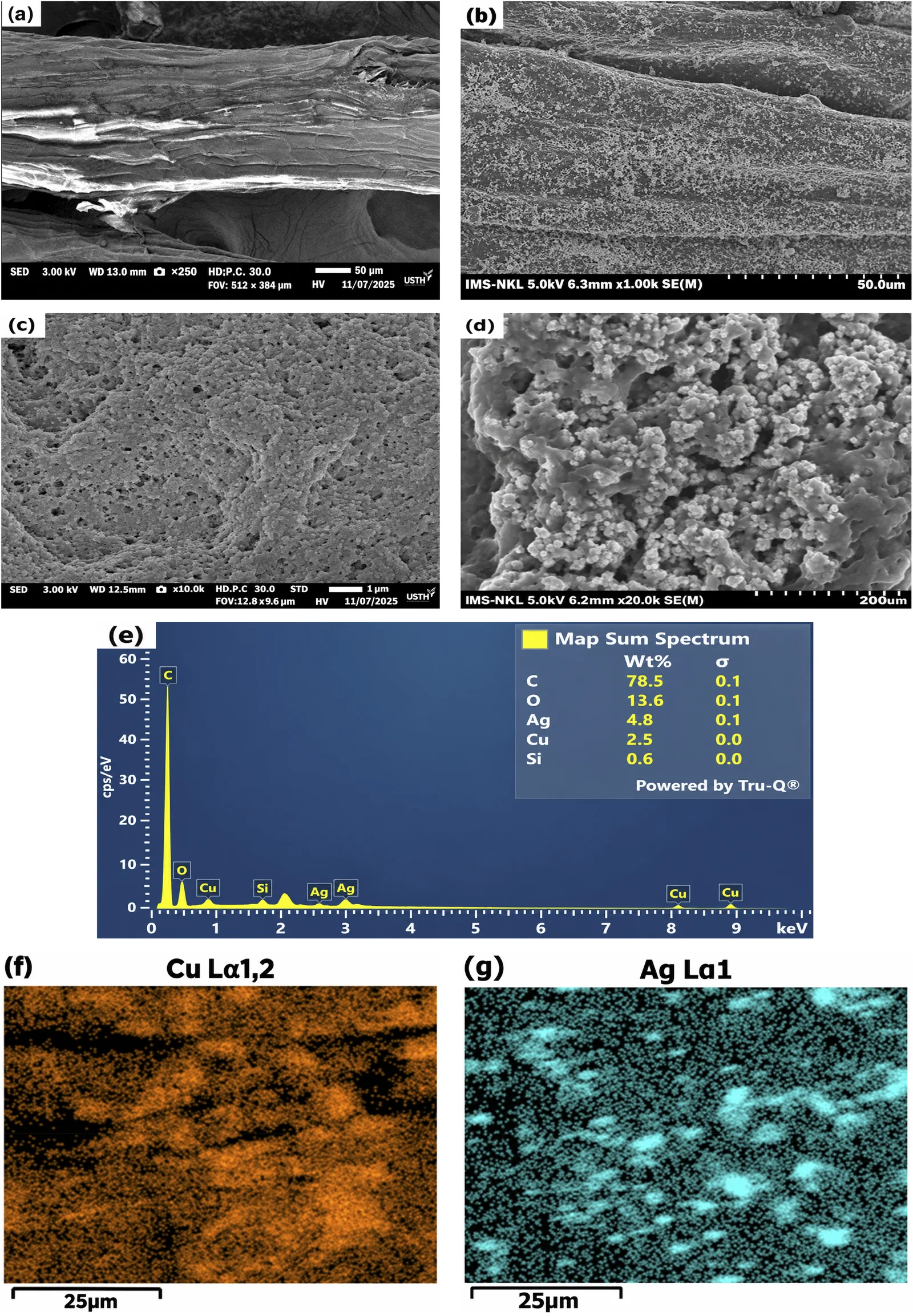
SEM-EDX characterization of the Cu_2_O–Ag@ACR/LF-3D monolith: (a–d) SEM images of the loofah scaffold and the composite surface at increasing magnifications; (e) EDX spectrum and semi-quantitative elemental composition; and (f, g) EDX elemental maps of Cu and Ag, respectively.

Pristine LF-3D exhibits the characteristic fibrous and porous morphology of a lignocellulosic loofah scaffold, with relatively smooth surface regions and open voids between adjacent structural elements, consistent with previous SEM observations of *Luffa cylindrica*-based materials.^16,58^ After Cu_2_O–Ag deposition and acrylic-assisted immobilization, the fiber surface becomes markedly rougher and is covered by particulate domains (Fig. 3b–d). At low magnification, the interconnected porous framework of the loofah scaffold remains clearly visible, indicating preservation of the overall monolithic architecture after immobilization. At higher magnification, dense granular and aggregated regions coexist with smaller open pores, revealing extensive but spatially non-uniform surface coverage. Similar particle-decorated morphologies have been reported for supported Cu_2_O- and Ag-containing composite systems.^10,32^

The EDX spectrum shows C and O signals associated predominantly with the lignocellulosic/acrylic matrix, together with Cu and Ag signals from the immobilized inorganic phase (Fig. 3e). No conductive carbon tape was used for specimen mounting; therefore, the strong C signal was not attributed to a carbon-tape background. In the analyzed surface region, the measured composition was 78.5 wt% C, 13.6 wt% O, 4.8 wt% Ag, and 2.5 wt% Cu, with a minor Si contribution of 0.6 wt%. Because EDX provides local and semi-quantitative elemental information, these values are interpreted only as representative of the analyzed surface region and not as the bulk composition of the monolith. Accordingly, the Cu and Ag signals are used primarily to support their local presence and spatial distribution within the analyzed surface region.^10,32^

Elemental mapping further shows that Cu and Ag are distributed across the analyzed surface area (Fig. 3f and g), supporting the presence of immobilized Cu- and Ag-containing domains on the loofah-based composite surface. Comparable mapping approaches have been used to examine the distribution of Cu-containing semiconductor phases on cellulose-based supports and Cu_2_O/Ag-containing substrates.^10,59^ Importantly, the present maps demonstrate surface co-occurrence of Cu and Ag but do not resolve their nanoscale contact geometry and therefore cannot, by themselves, establish a specific interfacial charge-transfer pathway.

N_2_ adsorption–desorption measurements were performed to examine the N_2_-accessible textural characteristics of the monoliths (Fig. 4). The isotherms showed features broadly resembling Type II behavior, although the low N_2_ uptake limited the confidence of this classification. The hysteresis features were insufficiently resolved to support assignment to a specific IUPAC hysteresis-loop type.

**Fig. 4:**
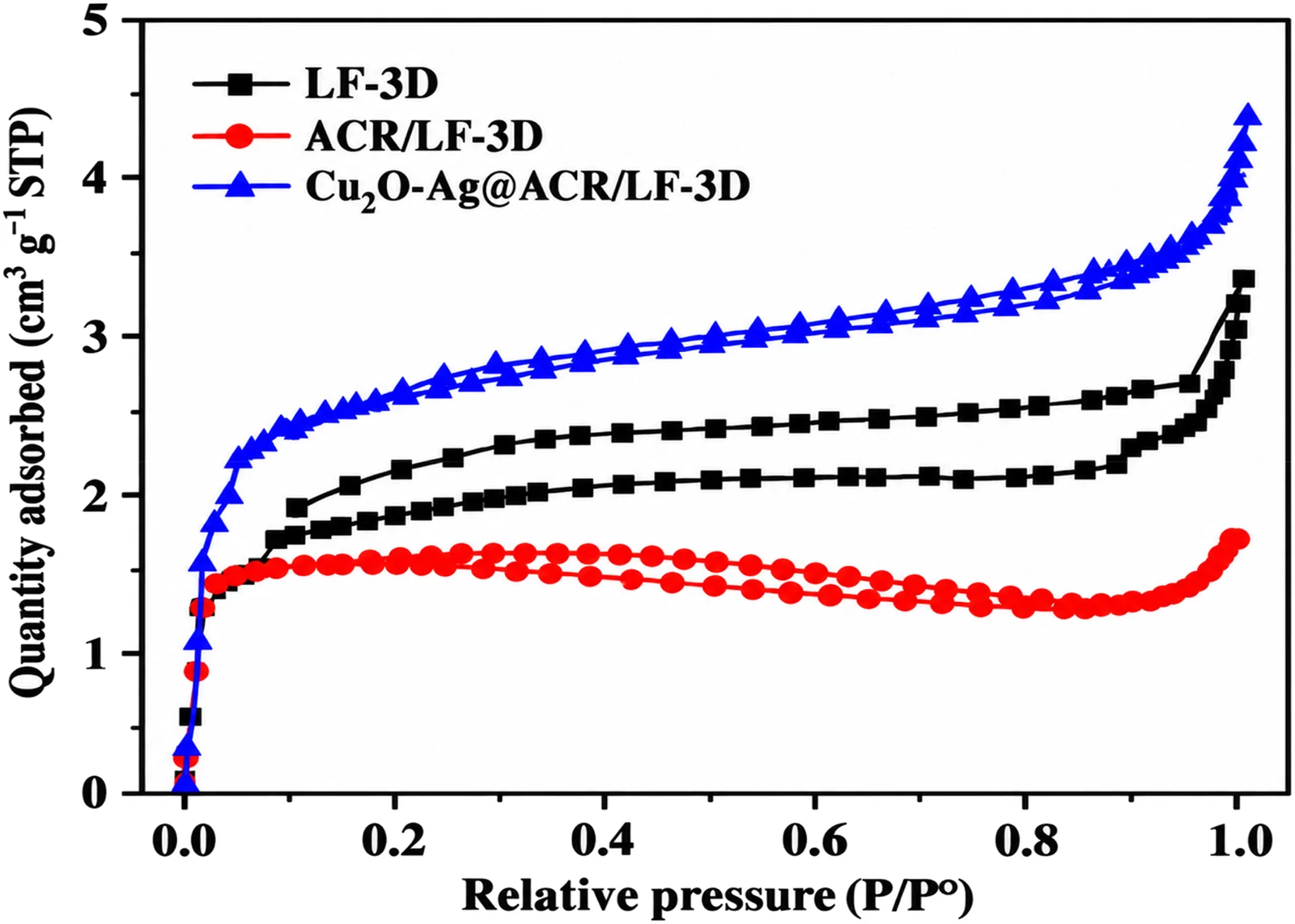
N_2_ adsorption–desorption isotherms of LF-3D, ACR/LF-3D, and Cu_2_O–Ag@ACR/LF-3D at 77.35 K.

The BET analysis yielded calculated surface-area values of 5.65, 4.21, and 8.03 m^2^ g^−1^ for LF-3D, ACR/LF-3D, and Cu_2_O–Ag@ACR/LF-3D, respectively. However, re-examination of the BET fitting intervals revealed that these values were sensitive to the selected relative-pressure ranges. They are therefore treated as tentative estimates and are not used to establish quantitative changes in specific surface area during monolith fabrication. Given the low N_2_ uptake and the uncertainty associated with the physisorption data, quantitative pore-size distributions were not considered sufficiently reliable for interpretation and are therefore not reported.

SEM observations showed that the interconnected macroscopic architecture was retained following acrylic modification and Cu_2_O–Ag immobilization, while EDX supported the local presence of Cu- and Ag-containing domains in the analyzed regions. These findings provide complementary morphological and local elemental information but do not establish quantitative pore characteristics.

### 3.2. Visible-light decolorization performance and operating-factor effects

Before evaluating the effects of the operating variables, component-resolved visible-light control experiments were performed using LF-3D, ACR/LF-3D, Ag@ACR/LF-3D, Cu_2_O@ACR/LF-3D, and the complete Cu_2_O–Ag@ACR/LF-3D monolith under otherwise identical irradiation conditions. The UV-vis spectra after 180 min of irradiation are compared with the initial irradiation-stage spectrum in Fig. 5a.

**Fig. 5:**
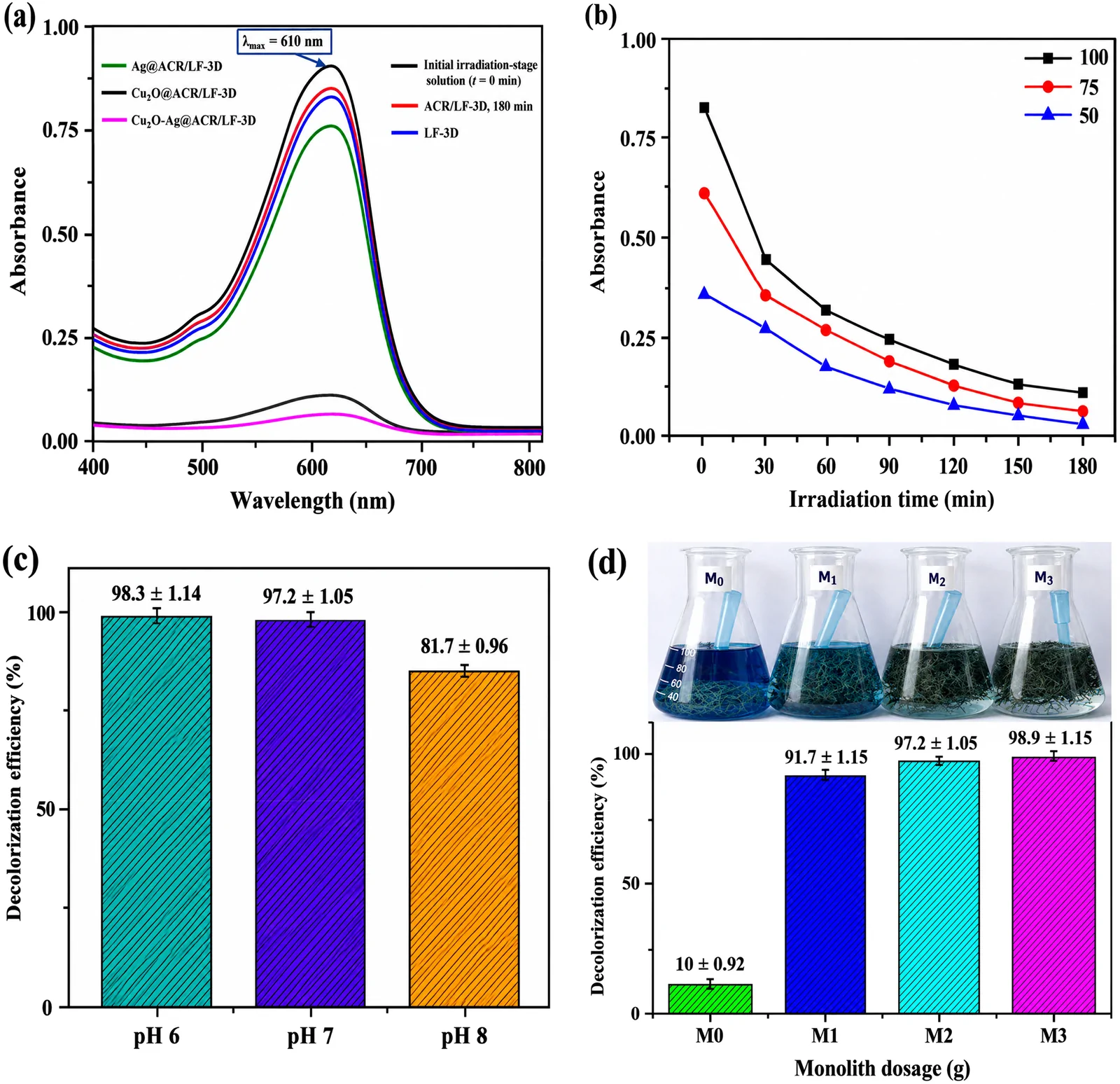
Visible-light decolorization performance of Cu_2_O–Ag@ACR/LF-3D: (a) component-resolved controls after 180 min of irradiation; (b) absorbance profiles at different initial dye concentrations; (c) effect of solution pH; and (d) effect of monolith dosage. M0 denotes the LF-3D control (0.75 g), whereas M1–M3 represent Cu_2_O–Ag@ACR/LF-3D dosages of 0.50, 0.75, and 1.00 g, respectively. Absorbance data in (a and b) were measured after two-fold dilution and are presented without back-correction.

The spectra represent directly measured absorbance values of two-fold diluted samples without back-correction to the undiluted samples. All samples retained the characteristic dye absorption band centered at approximately 610 nm, but markedly different decreases in absorbance were observed among the material configurations. The corresponding *H*_photo_, 180 values were 7.62, 7.00, 17.00, 63.61, and 94.66% for LF-3D, ACR/LF-3D, Ag@ACR/LF-3D, Cu_2_O@ACR/LF-3D, and Cu_2_O–Ag@ACR/LF-3D, respectively. The similarly low responses of LF-3D and ACR/LF-3D indicate that neither the loofah scaffold nor the acrylic modification alone accounts for the pronounced visible-light response. Ag@ACR/LF-3D exhibited only modest photodecolorization, whereas Cu_2_O@ACR/LF-3D showed substantially greater activity, indicating that Cu_2_O provides the dominant visible-light-responsive contribution among the tested component configurations. The further increase observed for Cu_2_O–Ag@ACR/LF-3D indicates a beneficial contribution associated with Ag incorporation, consistent with enhanced photocatalytic behavior reported for other Cu_2_O-based Ag-containing heterojunctions.^60^ These component-resolved controls establish differences in visible-light performance but do not, by themselves, demonstrate a specific Cu_2_O–Ag interfacial charge-transfer pathway.

Following the component-resolved control experiments, the effects of initial dye concentration, solution pH, and monolith dosage on the visible-light decolorization performance of Cu_2_O–Ag@ACR/LF-3D were examined, as summarized in Fig. 5b–d.

As the initial dye concentration increased from 50 to 100 mg L^−1^, the measured absorbance remained progressively higher during irradiation (Fig. 5b), indicating less favorable decolorization at higher dye loading. The plotted absorbance values were obtained directly from two-fold-diluted aliquots without back-correction, whereas dye concentrations in the original samples were calculated using the calibration relationship and the corresponding dilution factors. This behavior is consistent with stronger optical attenuation and increased competition for accessible adsorption/photoactive domains at higher dye concentrations.^2,9^ Within the investigated range, the response therefore reflects a balance between interfacial dye availability and effective photon access to the structured monolith.

Solution pH also strongly affected decolorization efficiency (Fig. 5c). The highest efficiencies were obtained at pH 6 and 7, reaching 98.3% and 97.2%, respectively, whereas the value decreased to 81.7% at pH 8. The lower performance under more alkaline conditions may reflect changes in dye ionization, interfacial charge conditions, and dye-surface interactions at the lignocellulosic/acrylic interface.^2,17,18^ The pronounced pH dependence is consistent with previous observations for loofah-based dye-removal systems, in which surface chemistry and dye speciation govern interfacial uptake.^22^

Increasing the Cu_2_O–Ag@ACR/LF-3D dosage from 0.50 to 0.75 and 1.00 g increased the decolorization efficiency from 91.7% to 97.2% and 98.9%, respectively (Fig. 5d). The improvement is consistent with a greater number of solution-accessible adsorption and photoactive domains at higher monolith dosage. However, the limited increase between 0.75 and 1.00 g indicates a diminishing dosage effect, suggesting that nominal catalyst availability increasingly becomes constrained by photon accessibility and interfacial mass transfer within the structured system.^39,40^

An unsupported Cu_2_O–Ag powder was not included as a direct control because the present study was designed to resolve component contributions within a fixed monolithic architecture. A dispersed powder would differ simultaneously in catalyst dispersion, illuminated surface distribution, solid–liquid contact, hydrodynamics, and recovery mode, thereby preventing an equivalent component-level comparison with the immobilized monolith. In general, dispersed photocatalysts can offer high external surface accessibility, whereas immobilized three-dimensional systems simplify catalyst recovery and repeated handling but may introduce photon-transport and mass-transfer limitations.^7,11,39,40^ The present study therefore focuses on the structure–function–stability behavior of the recoverable monolithic configuration rather than on benchmarking it against a non-equivalent dispersed powder system.

Overall, the single-factor experiments show that decolorization performance is governed by the coupled effects of dye loading, interfacial conditions, and the availability of illuminated photoactive domains within the monolithic architecture. These observations provided the basis for the subsequent apparent kinetic analysis and multivariable optimization.

### 3.3. Apparent visible-light photodecolorization kinetics

The irradiation-stage decolorization data were fitted using the pseudo-first-order model described in Section 2.5.3. Apparent first-order kinetic treatments are commonly used as comparative descriptions of heterogeneous photocatalytic dye degradation.^10,12^ The coefficients of determination (*R*^2^) ranged from 0.9752 to 0.9988, indicating satisfactory agreement between the model and the experimental data over the investigated irradiation period. Accordingly, the fitted *k*_app_ values were interpreted only as comparative kinetic descriptors under the specified irradiation conditions and not as intrinsic photocatalytic rate constants. The fitted kinetic equations, apparent rate constants, and corresponding *R*^2^ values are summarized in Table 1.

**Table 1:** Apparent pseudo-first-order kinetic parameters for visible-light decolorization by Cu_2_O–Ag@ACR/LF-3D

Monolith dosage (g)	*C*_0_ (mg L^−1^)	Kinetic equation	*K*_app_ (min^−1^)	*R* ^2^
Cu_2_O–Ag@ACR/LF-3D 0.75	50	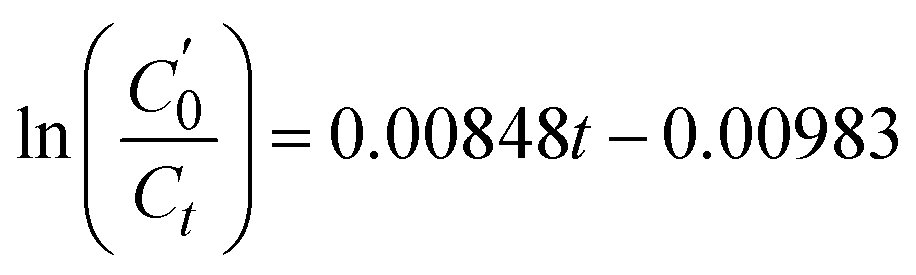	8.48 × 10^−3^	0.9988
Cu_2_O–Ag@ACR/LF-3D 0.75	75	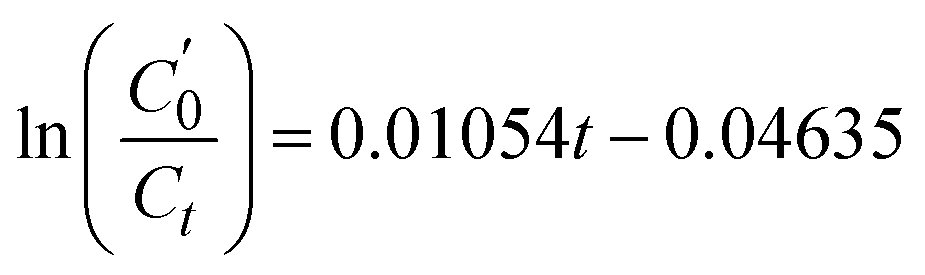	1.05 × 10^−2^	0.9824
Cu_2_O–Ag@ACR/LF-3D 0.75	100	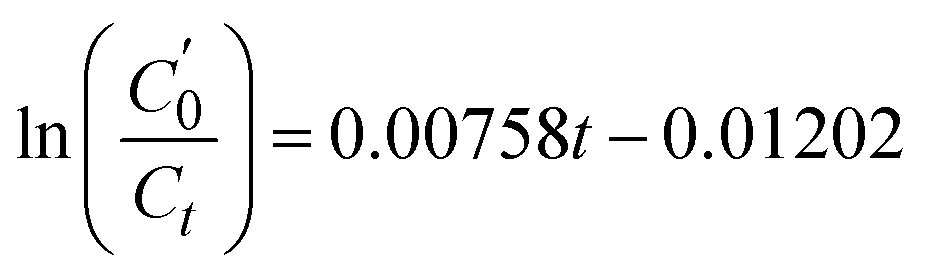	7.58 × 10^−3^	0.9758
Cu_2_O–Ag@ACR/LF-3D 0.5	75	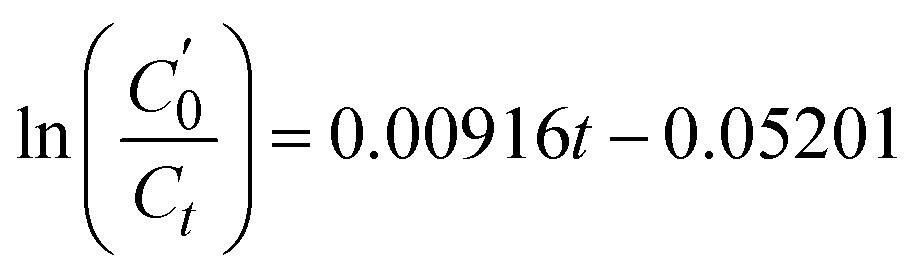	9.16 × 10^−3^	0.9752
Cu_2_O–Ag@ACR/LF-3D 1.0	75	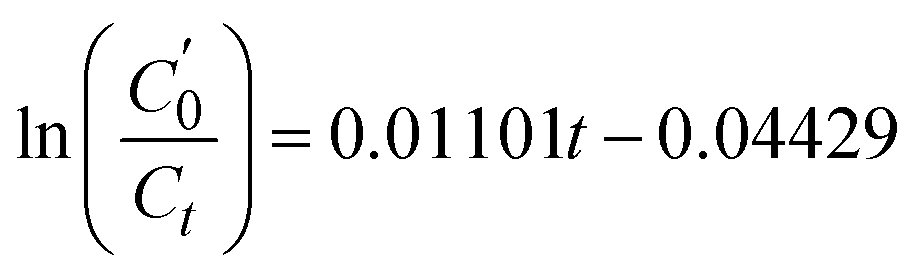	1.10 × 10^−2^	0.9881

At a fixed monolith dosage of 0.75 g, *k*_app_ values of 8.48 × 10^−3^, 1.05 × 10^−2^, and 7.58 × 10^−3^ min^−1^ were obtained at initial dye concentrations of 50, 75, and 100 mg L^−1^, respectively. The concentration dependence was therefore non-monotonic, with the highest apparent irradiation-stage rate observed at 75 mg L^−1^. The decrease at 100 mg L^−1^ is consistent with stronger optical attenuation and increased competition for accessible adsorption/photoactive domains at higher dye loading.^12^

The lower *k*_app_ observed at 50 mg L^−1^ indicates that the irradiation-stage response was not governed by optical attenuation alone. Because Cu_2_O–Ag@ACR/LF-3D combines an adsorption-active lignocellulosic/acrylic interface with immobilized photoactive domains, the observed concentration dependence likely reflects a balance among interfacial dye availability, accessible active domains, and photon penetration. Concentration-dependent adsorption behavior has likewise been reported for Cu_2_O-containing and lignocellulosic dye-removal systems.^31^ The maximum *k*_app_ at 75 mg L^−1^ should therefore be regarded as an operating-point response within the investigated range rather than as evidence of a unique mechanistic optimum.

At *C*_0_ = 75 mg L^−1^, increasing the monolith dosage from 0.50 to 0.75 and 1.00 g increased *k*_app_ from 9.16 × 10^−3^ to 1.05 × 10^−2^ and 1.10 × 10^−2^ min^−1^, respectively. The limited increase between 0.75 and 1.00 g (approximately 4.5%) indicates a diminishing dosage effect, consistent with the progressive constraint of additional catalyst availability by photon accessibility and interfacial mass transfer in structured photocatalytic systems.^7,10,12^

The fitted intercepts ranged from −0.00983 to −0.05201 and remained close to zero. These deviations may reflect uncertainty in determining 
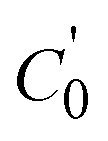
, sampling at the onset of irradiation, and continued interfacial equilibration during the early irradiation period. Their limited magnitude supports the use of 
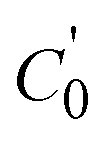
 as the practical reference concentration for comparative irradiation-stage kinetic analysis.

### 3.4. Optimization of the photocatalytic process

#### 3.4.1. Box–Behnken design and experimental responses

The visible-light decolorization efficiency, *H*_total_, of Cu_2_O–Ag@ACR/LF-3D was evaluated using a three-factor Box–Behnken design within the response surface methodology (RSM) framework. The investigated variables were solution pH (*A*, 5.5–6.5), initial dye concentration (*B*, 65–75 mg L^−1^), and monolith dosage (*C*, 0.65–0.75 g). A total of 17 experimental runs, including five center-point replicates, were performed to estimate pure error and assess experimental repeatability^52–54^ (Table 2).

**Table 2:** Box–Behnken design matrix and experimental values for visible-light decolorization by Cu_2_O–Ag@ACR/LF-3D

Run	Experimental conditions for reactive dye treatment			Decolorization efficiency *H*_total_ (%)
	pH (*A*)	Initial dye concentration (mg L^−1^) (*B*)	Material dosage (g) (*C*)	
1	6.5	70	0.75	60.34
2	6.0	70	0.70	96.04
3	6.0	65	0.65	74.83
4	6.0	70	0.70	96.79
5	5.5	70	0.75	93.69
6	5.5	70	0.65	86.43
7	5.5	65	0.70	82.56
8	5.5	75	0.70	85.67
9	6.5	65	0.70	53.65
10	6.0	75	0.65	79.52
11	6.5	75	0.70	48.73
12	6.5	70	0.65	55.68
13	6.0	70	0.70	97.03
14	6.0	70	0.70	96.54
15	6.0	70	0.70	98.98
16	6.0	75	0.75	83.01
17	6.0	65	0.75	88.67

Across the 17 runs, *H*_total_ varied from 48.73% to 98.98%, demonstrating a pronounced dependence on the combined operating conditions within the investigated design space. The lowest response occurred at pH 6.5, an initial dye concentration of 75 mg L^−1^, and a monolith dosage of 0.70 g, whereas the highest response was obtained at the center condition of pH 6.0, 70 mg L^−1^, and 0.70 g. The five center-point runs yielded decolorization efficiencies of 96.04–98.98%, indicating good repeatability and providing a reliable estimate of pure error for subsequent model assessment.

The broad response variation observed over the relatively narrow factor ranges indicates that decolorization is governed by coupled and nonlinear effects rather than by any single operating variable. This behavior justifies multivariable response-surface analysis of the structured Cu_2_O–Ag@ACR/LF-3D system.^52,54^

#### 3.4.2. ANOVA and adequacy of the quadratic model

The quadratic model was highly significant, with *F* = 377.35 and *p* < 0.0001 (Table 3). In contrast, the lack of fit was not significant relative to the pure error (*F* = 1.09, *p* = 0.4506), indicating no evidence of systematic model inadequacy within the investigated design space.

**Table 3:** ANOVA for the quadratic model describing visible-light decolorization by Cu_2_O–Ag@ACR/LF-3D

Source	Sum of squares	*df*	Mean square	*F*-value	*p*-value	Remarks
Model	4463.19	9	495.91	377.35	<0.0001	Significant
*A*-solution pH	2110.88	1	2110.88	1606.23	<0.0001	Significant
*B*-initial dye concentration (mg L^−1^)	0.9660	1	0.9660	0.7351	0.4196	Not significant
*C*-monolith dosage (g)	106.95	1	106.95	81.38	<0.0001	Significant
*AB*	16.12	1	16.12	12.27	0.0100	Significant
*AC*	1.69	1	1.69	1.29	0.2941	Not significant
*BC*	26.78	1	26.78	20.38	0.0028	Significant
*A* ^2^	1432.96	1	1432.96	1090.39	<0.0001	Significant
*B* ^2^	507.21	1	507.21	385.95	<0.0001	Significant
*C* ^2^	88.82	1	88.82	67.59	<0.0001	Significant
Residual	9.20	7	1.31	—	—	—
Lack of fit	4.13	3	1.38	1.09	0.4506	Not significant
Pure error	5.07	4	1.27	—	—	—
Cor. total	4472.38	16	—	—	—	—
*R* ^2^	0.9979					
Adjusted *R*^2^	0.9953					
Predicted *R*^2^	0.9835					

Among the linear terms, solution pH (*A*) exerted the strongest effect on *H*_total_ (*F* = 1606.23, *p* < 0.0001), followed by monolith dosage (*C*; *F* = 81.38, *p* < 0.0001). The linear contribution of initial dye concentration (*B*) was not significant (*p* = 0.4196). However, all three quadratic terms, *A*^2^, *B*^2^, and *C*^2^, were highly significant (*p* < 0.0001), revealing pronounced curvature in the response surface. In particular, the combination of a non-significant linear *B* term and a highly significant *B*^2^ term shows that the influence of dye concentration was predominantly nonlinear rather than negligible. This behavior is consistent with the competing effects of interfacial dye availability, optical attenuation, and access to photoactive domains within the structured monolith.^52,54^

The interaction terms *AB* and *BC* were significant (*p* = 0.0100 and 0.0028, respectively), whereas *AC* was not (*p* = 0.2941). Thus, the influence of dye concentration depended on both solution pH and monolith dosage, while no statistically significant pH-dosage interaction was detected within the investigated ranges.

The model showed strong goodness of fit, with *R*^2^ = 0.9979, adjusted *R*^2^ = 0.9953, and predicted *R*^2^ = 0.9835. Together with the non-significant lack of fit, these statistics support reliable interpolation within the experimental domain. The coefficient of variation was 1.41%, and the Adeq Precision value was 54.6185, indicating adequate experimental precision and signal-to-noise ratio for response-surface analysis.

Overall, solution pH was the dominant operating factor, monolith dosage also contributed significantly, and initial dye concentration exhibited a predominantly nonlinear response with significant coupling to both pH and dosage. The resulting quadratic model was therefore suitable for interpretation and numerical optimization within the investigated design space.

#### 3.4.3. Model diagnostics and response-surface analysis

The diagnostic plots and representative response surfaces for *H*_total_ are shown in Fig. 6. The predicted values closely followed the experimental responses along the 1 : 1 line (Fig. 6a), consistent with the high *R*^2^ value of 0.9979. The externally studentized residuals were randomly distributed around zero and remained within the diagnostic limits of approximately ±4.82 (Fig. 6b). Together with the non-significant lack of fit, these observations support the adequacy of the quadratic model within the investigated design space.

**Fig. 6:**
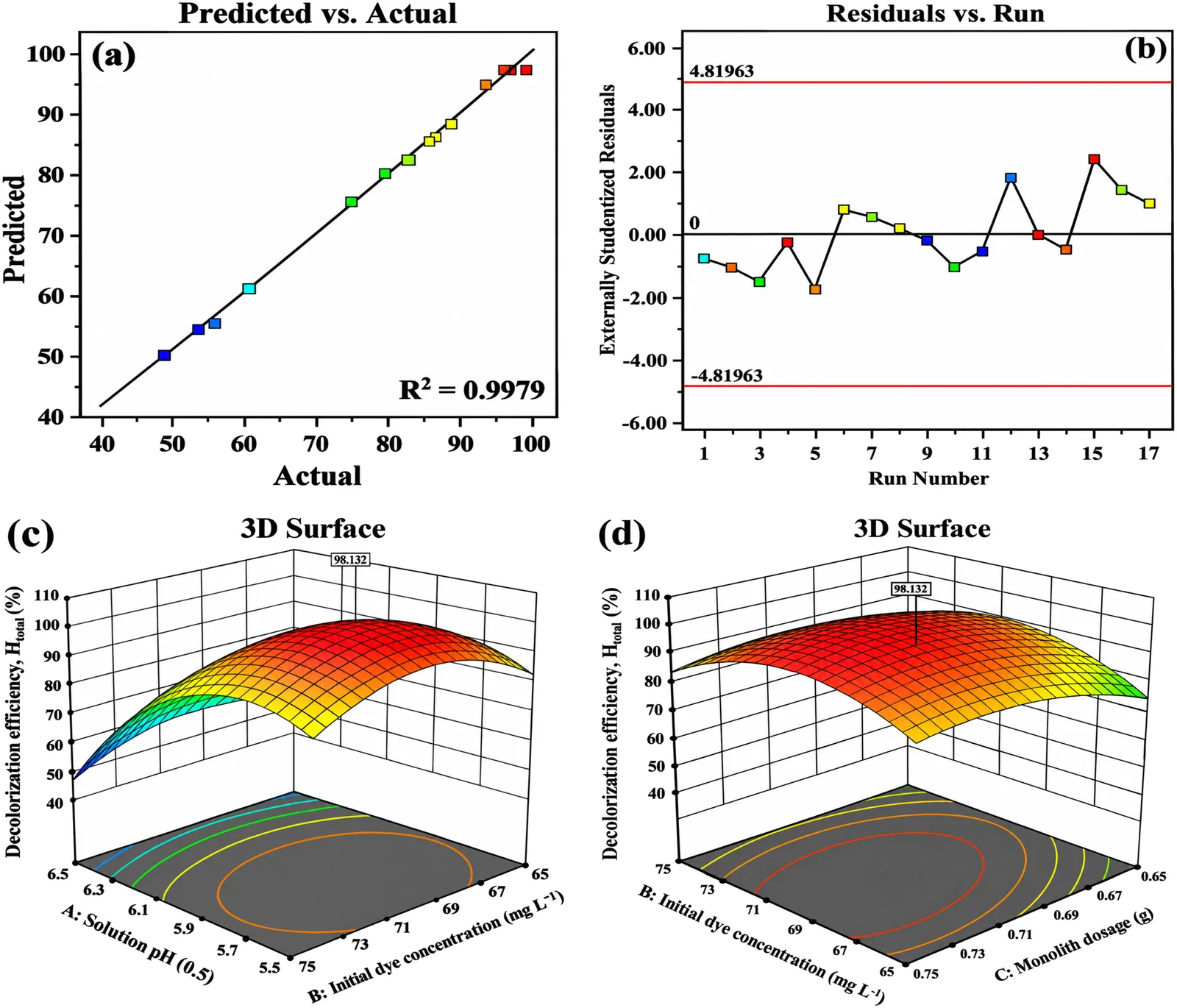
RSM diagnostics and response surfaces for visible-light decolorization by Cu_2_O–Ag@ACR/LF-3D: (a) predicted *versus* experimental values, (b) externally studentized residuals *versus* run order, (c) effects of solution pH and initial dye concentration, and (d) effects of initial dye concentration and monolith dosage.

The pH-concentration response surface exhibits pronounced curvature, with a high-response region near pH 6.0 and an intermediate dye concentration (Fig. 6c). The response decreases markedly toward pH 6.5, while the effect of dye concentration varies with pH, consistent with the significant *A*, *A*^2^, *B*^2^, and *AB* terms identified by ANOVA.

At the center pH level, the concentration-dosage response surface shows that increasing monolith dosage generally improves *H*_total_, whereas the influence of dye concentration remains nonlinear (Fig. 6d). The significant *BC* interaction (*p* = 0.0028) indicates that the dosage effect depends on dye concentration within the investigated range. This behavior is consistent with a balance between solution-accessible adsorption/photoactive domains and concentration-dependent photon attenuation within the structured monolith.

Overall, the response surfaces identify a relatively narrow high-performance region near the center of the investigated design space, providing the basis for subsequent numerical optimization.

#### 3.4.4. Experimental validation and operational stability

Numerical optimization of the quadratic model predicted a maximum *H*_total_ of 98.14% at pH 5.94, an initial dye concentration of 68.07 mg L^−1^, and a Cu_2_O–Ag@ACR/LF-3D dosage of 0.71 g. The predicted optimum lies within the investigated design space and within the high-response region identified from the response surfaces rather than at a factor boundary. A confirmation experiment conducted under these conditions yielded an experimental decolorization efficiency of 96.43%, corresponding to a relative deviation of 1.74% from the predicted value. This close agreement supports the reliability of the quadratic model for interpolation and numerical optimization within the investigated factor ranges. Because the optimum falls within the predefined experimental domain, these validated conditions were subsequently used to evaluate monolith performance and operational stability. Extrapolation beyond the investigated ranges would require additional experimental verification.

To evaluate treatment performance beyond chromophore attenuation, TOC was additionally determined for the optimized Cu_2_O–Ag@ACR/LF-3D system at the beginning of the irradiation stage and after 180 min of visible-light exposure. The TOC decreased by approximately 62%, corresponding to 
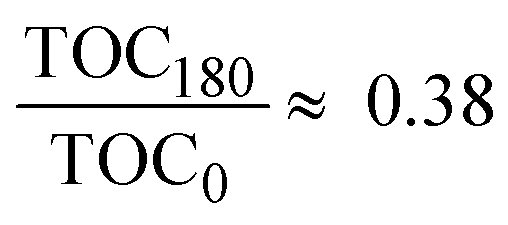
, whereas the corresponding decolorization efficiency reached 96.43%. The markedly lower TOC reduction compared with chromophore decolorization indicates that loss of visible color proceeded more rapidly than complete oxidation of the organic carbon. The treatment therefore achieved substantial but incomplete mineralization. Similar differences between pollutant degradation and TOC removal have been reported for Cu_2_O-containing photocatalytic systems. CeO_2_/Cu_2_O showed approximately 68.98% TOC removal after visible-light irradiation,^61^ whereas ZnO–Cu_2_O–chitosan achieved 74.86% TOC removal for methyl orange despite nearly complete degradation.^62^ A Cu@Cu_2_O@CuO-microalgae photocatalyst likewise exhibited lower mineralization than pollutant degradation at intermediate irradiation times, confirming that mineralization can proceed through multiple oxidation steps.^63^ Because transformation products and their toxicity were not directly characterized in the present study, the TOC results should not be interpreted as evidence of complete detoxification or absence of potentially persistent intermediates.

The monolith was further evaluated for reusability, system-specific contributions, and reactive-species involvement under visible-light irradiation (Fig. 7).

**Fig. 7:**
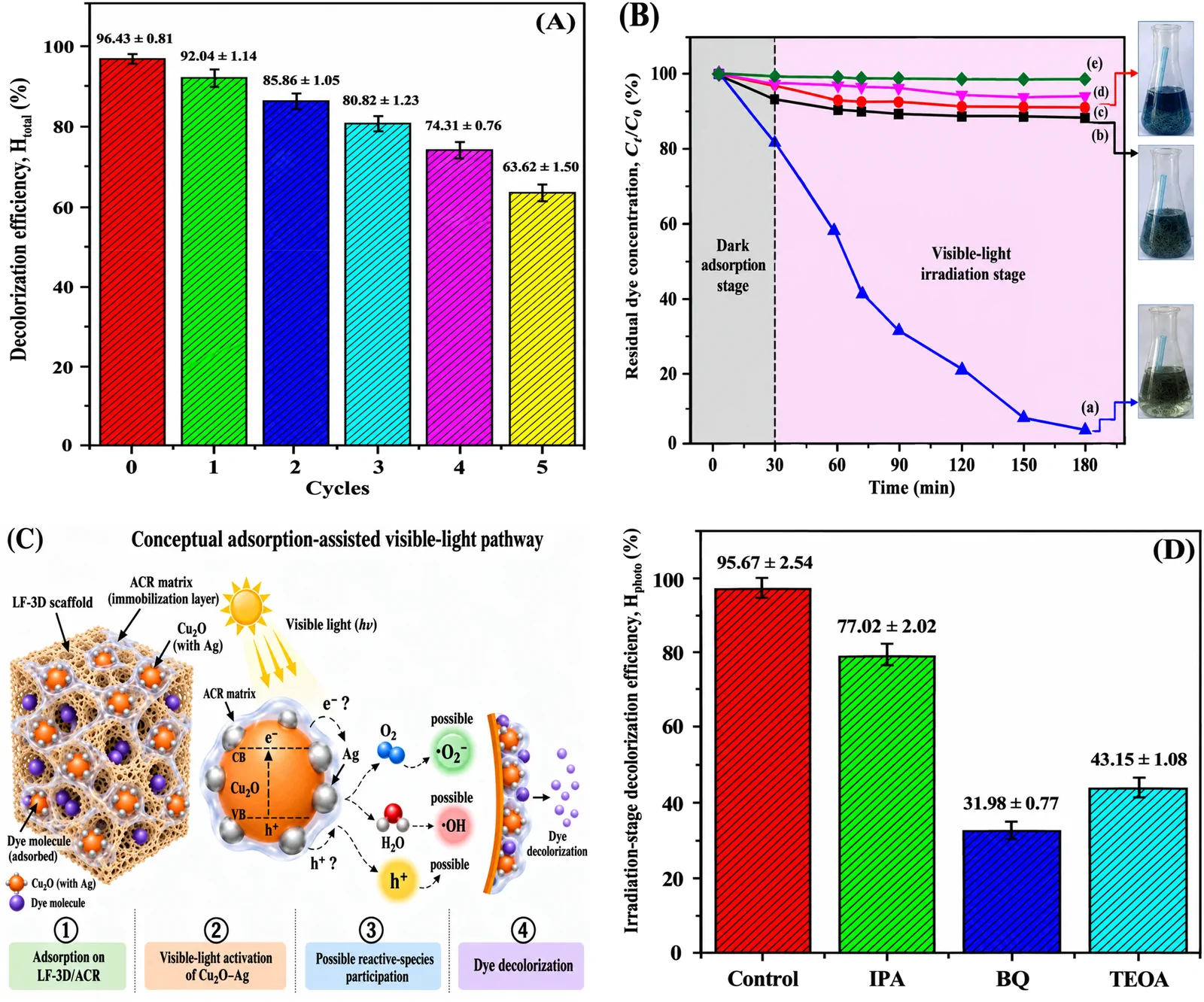
Reusability, controls, and mechanistic assessment of Cu_2_O–Ag@ACR/LF-3D: (A) reuse performance; (B) residual dye concentration for the monolith and controls; (C) literature-supported conceptual adsorption-assisted visible-light pathway; and (D) reactive-species trapping after 180 min of visible-light irradiation.

The reusability of Cu_2_O–Ag@ACR/LF-3D was then evaluated under the experimentally validated conditions. The fresh monolith exhibited a decolorization efficiency of 96.43%, which decreased to 92.04%, 85.86%, 80.82%, 74.31%, and 63.62% after reuse cycles 1–5, respectively (Fig. 7A). Thus, after five reuse cycles, the monolith retained approximately 65.97% of its initial decolorization performance. Although activity progressively declined, the monolith remained physically recoverable throughout repeated operation. Similar cycle-dependent losses have been reported for supported Cu_2_O–Ag-containing systems and have been associated with incomplete desorption, reduced accessibility of surface-active domains, and changes occurring during repeated aqueous operation.^10,34^

The progressive activity loss may reflect surface masking by retained dye or transformation products, reduced accessibility of Cu_2_O–Ag-containing domains, and possible changes in near-surface Cu species during repeated aqueous operation. These effects could reduce interfacial accessibility without requiring macroscopic collapse of the LF-3D scaffold. The relative contributions of these processes are evaluated further through post-cycle SEM-EDX and solution-phase AAS in Section 3.5.2.

The expanded control experiments distinguish the responses of the complete Cu_2_O–Ag@ACR/LF-3D system, the supporting matrices, the leached solution, and direct photolysis (Fig. 7B). At 180 min, the normalized residual dye concentrations 
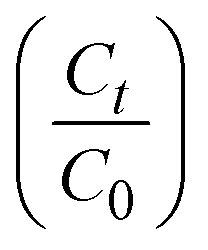
 were 3.56, 90.00, 91.66, 92.89, and 98.37% for Cu_2_O–Ag@ACR/LF-3D, LF-3D, ACR/LF-3D, leachate-only, and photolysis, respectively. The corresponding overall apparent dye-removal values were 96.44, 10.00, 8.34, 7.11, and 1.63%, respectively. The leachate-only system exhibited a slightly greater response than direct photolysis, indicating that dissolved species released from the monolith may contribute to dye decolorization to a limited extent. However, during the visible-light irradiation stage, 
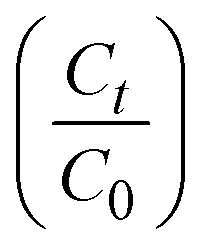
 decreased only from 97.96 to 92.89% for the leachate-only system, compared with a decrease from 82.22 to 3.56% for the complete Cu_2_O–Ag@ACR/LF-3D system. The leached solution therefore did not reproduce the pronounced response of the intact monolith. These results indicate that the predominant contribution is associated with the immobilized monolithic phase, although a minor homogeneous contribution from dissolved species cannot be excluded. This behavior is consistent with the adsorption–photocatalysis coupling reported for supported Cu_2_O-containing systems.^10^

The scavenger experiments provide indirect evidence for the possible involvement of reactive species in visible-light-driven photodecolorization (Fig. 7D). After 180 min of irradiation, the decolorization efficiency was 95.67% for the control, compared with 77.02%, 31.98%, and 43.15% in the presence of isopropanol (IPA), *p*-benzoquinone (BQ), and triethanolamine (TEOA), respectively. The stronger inhibition by BQ and TEOA suggests contributions from superoxide-related pathways and photogenerated holes, respectively, whereas the smaller effect of IPA suggests a comparatively less pronounced role for hydroxyl radicals under the tested conditions. These interpretations remain tentative because of the limited selectivity and possible secondary effects of the scavengers. Based on these findings and relevant literature,^61–63^Fig. 7C presents a conceptual model of adsorption-assisted visible-light photodecolorization. The available evidence does not establish the direction of interfacial charge transfer, the specific electronic interactions between Cu_2_O- and Ag-containing domains, or changes in their chemical states during operation. Accordingly, the proposed pathway should be regarded as a mechanistic hypothesis rather than an experimentally verified interfacial charge-transfer mechanism.

### 3.5. Complementary modeling and post-cycle material stability

#### 3.5.1. Complementary ANN assessment of the RSM-defined response region

The complementary ANN model reproduced the overall experimental response pattern, yielding *R*^2^ = 0.9722, RMSE = 1.945, MAE = 1.467, and a maximum absolute error of 6.168 percentage points. The parity comparison showed that the ANN predictions generally followed the experimental trend, although with greater dispersion than the quadratic RSM predictions. Similar complementary use of ANN and RSM has been reported for nonlinear adsorption and related water-treatment systems.^52,55^

Because the ANN was developed using the same 17-run BBD dataset as the RSM model, these metrics represent model-data agreement rather than independent predictive validation. Accordingly, the ANN was used only as a complementary nonlinear consistency assessment of the RSM-defined response region, while RSM remained the primary framework for statistical interpretation, numerical optimization, and experimental confirmation.

#### 3.5.2. Post-cycle surface evolution and active-phase retention

Post-cycle SEM-EDX and solution-phase AAS analyses were used to evaluate surface evolution and active-phase stability during repeated operation (Fig. 8). The fresh Cu_2_O–Ag@ACR/LF-3D monolith exhibited a porous, particle-decorated surface with numerous deposited domains (Fig. 8a and b). After cycling, the spent surface appeared locally smoother and less densely decorated, while the underlying porous framework remained evident (Fig. 8c and d). These observations indicate preservation of the three-dimensional scaffold together with local surface modification, which may involve partial masking, redistribution, or restructuring of the deposited phase during repeated aqueous treatment. Similar preservation of luffa-derived porous frameworks after modification and aqueous use has been reported.^29^

**Fig. 8:**
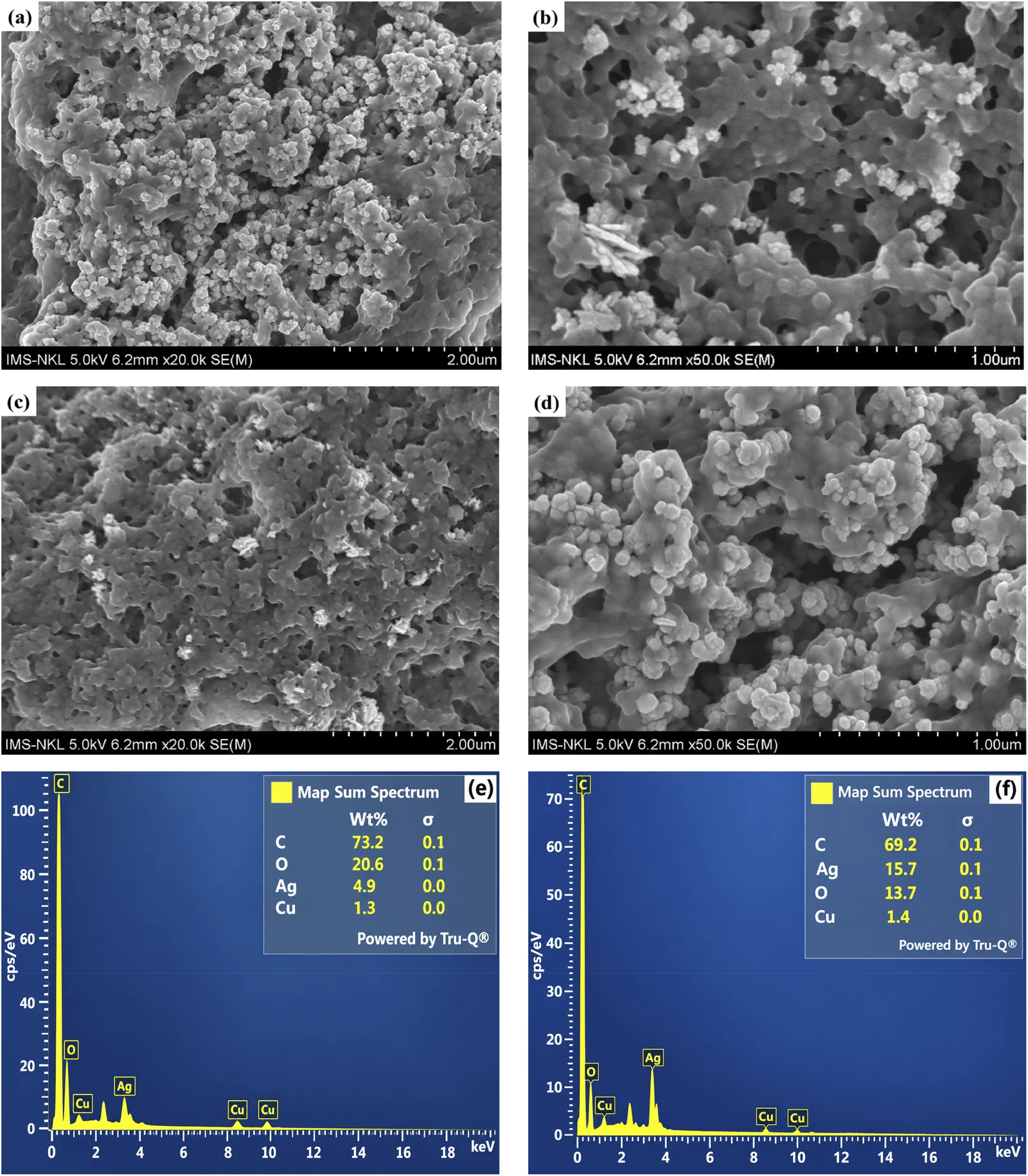
Post-cycle surface evolution and elemental characteristics of Cu_2_O–Ag@ACR/LF-3D: SEM images of the fresh monolith at (a) 20 000× and (b) 50 000× and the spent monolith at (c) 20 000× and (d) 50 000×; representative EDX spectra of the (e) fresh and (f) spent surfaces.

Representative EDX spectra showed that both Cu and Ag remained detectable on the monolith surface after repeated operation (Fig. 8e and f). However, the measured Cu and Ag fractions varied markedly among the analyzed regions of the fresh and spent samples, consistent with the heterogeneous elemental distribution observed before cycling. The EDX data were therefore interpreted only as local, semi-quantitative indicators of residual surface composition and not as quantitative measures of bulk metal retention or loss.

Solution-phase AAS provided complementary evidence of metal release during operation. After correction for dilution, dissolved Cu concentrations were 52.66, 60.52, and 88.64 mg L^−1^ for the three investigated samples, whereas the corresponding Ag concentrations were substantially lower at 0.07, 0.11, and 0.07 mg L^−1^, respectively. For the 200 mL working volume, these Cu concentrations correspond to approximately 10.53, 12.10, and 17.73 mg of dissolved Cu, respectively. Because the analyzed solutions were obtained from different monolith dosages rather than replicate measurements, the values were considered individually. Moreover, in the absence of a complete mass balance based on the initial bulk Cu and Ag inventories of the monoliths, the AAS results are interpreted as solution-phase metal release rather than as percentage loss of the immobilized phases.

Importantly, the measurable Cu release detected by AAS did not translate into a comparable activity of the dissolved phase. In the leachate-only control, 92.89% of the initial normalized dye concentration remained after 180 min, compared with only 3.56% for the intact Cu_2_O–Ag@ACR/LF-3D system. Direct photolysis retained 98.37%. Thus, although the slightly greater response of the leachate relative to photolysis indicates a limited contribution from dissolved/leached species, the control experiment does not support homogeneous catalysis as the dominant origin of the observed dye removal. Cu release therefore remains primarily an important active-phase-stability concern, while a minor homogeneous contribution cannot be excluded.

The present XRD, SEM-EDX, and AAS measurements do not resolve the near-surface oxidation states of Cu and Ag after repeated operation. Therefore, possible redistribution or changes in Cu(i)/Cu(ii) and Ag chemical states during cycling cannot be established from the present data and would require oxidation-state-sensitive surface analysis such as XPS. The absence of direct surface-sensitive chemical-state characterization before and after repeated operation limits conclusions regarding the oxidation states and chemical stability of the Cu- and Ag-containing domains. Further XPS and complementary surface and photoelectrochemical investigations would help clarify their chemical-state evolution and assess the proposed interfacial charge-transfer pathway.

Because SEM-EDX probes only selected near-surface regions, the persistence of Cu and Ag signals after cycling should not be interpreted as evidence of bulk metal retention. AAS provides evidence of solution-phase metal release, but the absence of a complete initial Cu and Ag inventory prevents quantification of the percentage loss of the immobilized phases.

Together, the post-cycle observations indicate that the porous LF-3D framework remained structurally recognizable, while the immobilized surface phase underwent local modification accompanied by measurable Cu release. The progressive decline in decolorization efficiency may be associated with reduced accessibility of active domains, surface masking, redistribution, and Cu loss, although their relative contributions cannot be determined from the available data. These results demonstrate physical recoverability and continued photocatalytic functionality but do not establish the chemical stability of the Cu- and Ag-containing domains after repeated operation. Active-phase retention, particularly Cu retention, therefore remains a principal materials-stability limitation of the present monolithic architecture.

Potential strategies for suppressing Cu loss include strengthening the interfacial anchoring of Cu-containing domains to the acrylic/loofah matrix and optimizing binder coverage and curing to improve particle retention without excessively blocking accessible photoactive sites, consistent with established approaches for photocatalyst immobilization on polymeric and lignocellulosic supports.^7,10,11^ Additional approaches, such as thin permeable protective or chelating interfacial layers, may also warrant investigation. These strategies remain directions for future material development and were not experimentally evaluated in the present study.

## 4. Conclusions

A recoverable Cu_2_O–Ag@ACR/LF-3D monolith was constructed through acrylic-assisted immobilization of Cu_2_O–Ag-containing domains on a three-dimensional loofah scaffold. The resulting architecture integrated a porous lignocellulosic framework, an acrylic immobilization interface, and visible-light-responsive domains while preserving the recognizable monolithic structure. This combination enabled adsorption-assisted visible-light photodecolorization, with the apparent kinetic response reflecting the coupled effects of interfacial dye availability, photon accessibility, and accessible photoactive domains.

Response surface methodology identified optimum conditions at pH 5.94, an initial dye concentration of 68.07 mg L^−1^, and a monolith dosage of 0.71 g. Under these conditions, the predicted decolorization efficiency of 98.14% agreed closely with the experimentally validated value of 96.43%. TOC decreased by approximately 62% after 180 min, indicating substantial but incomplete mineralization. Reactive-species trapping supported the participation of ˙O_2_^−^, h^+^, and ˙OH, with stronger contributions from ˙O_2_^−^ and h^+^-associated pathways, without establishing a definitive Cu_2_O–Ag interfacial charge-transfer pathway. The monolith remained readily recoverable during repeated operation, although the efficiency decreased to 63.62% after five reuse cycles.

Post-cycle SEM-EDX showed that Cu- and Ag-containing domains remained detectable at selected surface regions, whereas solution-phase AAS demonstrated measurable Cu release. Because SEM-EDX provides only local, semi-quantitative surface information, these observations should not be interpreted as evidence of bulk metal retention. Active-phase retention, particularly Cu retention, therefore remains a principal materials-stability limitation of the present architecture. Overall, the study establishes a structure–function–stability framework for integrating sorptive biomass interfaces and immobilized Cu_2_O–Ag-containing domains within a recoverable visible-light-responsive monolith. Further work should focus on improving active-phase retention and identifying residual transformation products.

## Author contributions

Xuan Thang Dam: conceptualization, methodology, supervision, formal analysis, data curation, visualization, writing – original draft, Writing – Review & Editing. Thi Thu Giang Pham: investigation. Thuy Van Ngo: investigation. Trong Tuan Nguyen: investigation. Truong Xuan Vuong: supervision, Writing – Review & Editing.

## Conflicts of interest

There are no conflicts to declare.

## Data Availability

The data supporting this article have been included as part of the main article and the supplementary information (SI). Supplementary information is available. See DOI: https://doi.org/10.1039/d6ra07776e.
